# Lipidation of pneumococcal proteins enables activation of human antigen-presenting cells and initiation of an adaptive immune response

**DOI:** 10.3389/fimmu.2024.1392316

**Published:** 2024-04-22

**Authors:** Antje D. Paulikat, Dominik Schwudke, Sven Hammerschmidt, Franziska Voß

**Affiliations:** ^1^ Department of Molecular Genetics and Infection Biology, Interfaculty Institute of Genetics and Functional Genomics, Center for Functional Genomics of Microbes, University of Greifswald, Greifswald, Germany; ^2^ Division of Bioanalytical Chemistry, Research Center Borstel - Leibniz Lung Center, Borstel, Germany; ^3^ German Center for Infection Research, Thematic Translational Unit Tuberculosis, Partner Site Hamburg-Lübeck-Borstel-Riems, Borstel, Germany; ^4^ German Center for Lung Research (DZL), Airway Research Center North (ARCN), Research Center Borstel, Leibniz Lung Center, Borstel, Germany

**Keywords:** *Streptococcus pneumoniae*, lipoproteins, lipidation, immune response, vaccine, antigen presenting cells, dendritic cells

## Abstract

*Streptococcus pneumoniae* remains a significant global threat, with existing vaccines having important limitations such as restricted serotype coverage and high manufacturing costs. Pneumococcal lipoproteins are emerging as promising vaccine candidates due to their surface exposure and conservation across various serotypes. While prior studies have explored their potential in mice, data in a human context and insights into the impact of the lipid moiety remain limited. In the present study, we examined the immunogenicity of two pneumococcal lipoproteins, DacB and MetQ, both in lipidated and non-lipidated versions, by stimulation of primary human immune cells. Immune responses were assessed by the expression of common surface markers for activation and maturation as well as cytokines released into the supernatant. Our findings indicate that in the case of MetQ lipidation was crucial for activation of human antigen-presenting cells such as dendritic cells and macrophages, while non-lipidated DacB demonstrated an intrinsic potential to induce an innate immune response. Nevertheless, immune responses to both proteins were enhanced by lipidation. Interestingly, following stimulation of dendritic cells with DacB, LipDacB and LipMetQ, cytokine levels of IL-6 and IL-23 were significantly increased, which are implicated in triggering potentially important Th17 cell responses. Furthermore, LipDacB and LipMetQ were able to induce proliferation of CD4+ T cells indicating their potential to induce an adaptive immune response. These findings contribute valuable insights into the immunogenic properties of pneumococcal lipoproteins, emphasizing their potential role in vaccine development against pneumococcal infections.

## 1 Introduction

*Streptococcus pneumoniae* (*S. pneumoniae*, the pneumococcus) is a leading cause of morbidity and mortality worldwide with at least 100 known different serotypes (1). A study covering the period from 1990 to 2016 reveals that, in the context of lower respiratory tract infections, pneumococci are responsible for more deaths than other bacteria (2). Young children, the elderly or immunocompromised individuals are at particular risk for pneumococcal infections (2–4). To date, several pneumococcal vaccines based on the outermost and serotype-determining polysaccharide capsule have been introduced. The most prominent representatives are the pneumococcal polysaccharide vaccine (PPV23) with purified polysaccharides against 23 serotypes or PCV13, a conjugate vaccine in which the polysaccharides of 13 serotypes have been coupled to a carrier protein to ensure immunogenicity in particularly vulnerable infants. Although both vaccines have been proven effective (5, 6), they have several important limitations. These are in particular restricted serotype coverage leading to increased incidence of infection with non-vaccine serotypes and high manufacturing costs (7, 8). In recent years, PCV13 has been extended by additional serotypes, leading to the introduction of PCV15 (Merck) and PCV20 (Pfizer). However, serotype coverage will remain an issue and the potentially improved efficacy of these vaccines in combating pneumococcal infections has yet to be demonstrated (9, 10). This has prompted research on new alternatives that could provide serotype-independent protection against pneumococcal infections.

Pneumococcal lipoproteins are promising candidates for vaccination. They represent the largest group of surface-associated proteins in pneumococci, are highly conserved across different serotypes and contribute to the bacterial fitness and pathogenesis (11–13). An example highlighting the promising nature of this approach is the availability of vaccines for meningococci based on lipoproteins (14). Several studies have already investigated the potential of different pneumococcal lipoproteins as new vaccine candidates (15–18). In addition, immunization of mice with pneumococcal membrane particles induced serotype-independent protection against invasive pneumococcal disease (IPD) that was mediated by the conserved lipoproteins MalX and PrsA (17). Furthermore, we previously demonstrated that immunization of mice with the l,d-carboxypeptidase DacB, the nucleoside-binding protein PnrA, and the methionine-binding protein MetQ, respectively, reduced pneumococcal colonization in the nasopharynx. This reduction correlated with high titers of Interleukin (IL)-17A (15, 16). Of note, lipidation of DacB and PnrA enhanced and modulated the humoral immune response to these recombinant proteins, as evidenced by increased antibody concentrations and elevated IgG2/IgG1 subclass ratios (16). Moreover, an interesting feature of lipoproteins is their ability to stimulate a Toll-like Receptor 2 (TLR 2)-driven immune response via their lipid moiety, underscoring their intrinsic adjuvant property (19–21).

In humans, antigen-presenting cells (APCs) play a key role in response to encountering pathogens and the subsequent development of immune memory cells. As part of the innate immune system, dendritic cells (DCs) as well as macrophages belong to the most efficient APC types in the human body. Both cell types express TLR2 on their surface, enabling them to recognize the lipid moiety of bacterial proteins and subsequently initiate an effective immune response. Mature DCs, particularly, possess the ability to activate naïve T cells (22) and can orchestrate the induced T cell response via the release of different cytokines (22–25). However, there are limited data on the effects of pneumococcal lipoproteins and their lipidation on the human immune system as most of the previous studies were performed in mice or solely focused on a whole-cell level (16, 20, 21).

In the present study, we aimed to investigate the impact of pneumococcal lipoproteins as potential vaccine candidates on human immune responses using different *in vitro* cell culture methods. On the one hand, human primary monocyte-derived dendritic cells (moDCs) were infected with lipoprotein diacylglyceryl transferase (Lgt) deficient pneumococci lacking lipid anchoring and cell membrane embedding of lipoproteins or Lgt expressing pneumococci to investigate the global effects of lipoproteins on the induced immune response. On the other hand, we stimulated APCs (moDCs and monocyte-derived macrophages (MDMs)) with pneumococcal lipoproteins DacB or MetQ and compared the activation profile of lipidated versus the non-lipidated protein versions based on the expression of different surface markers and secreted cytokines. Because DCs are the key players in initiating an adaptive immune response by activating T cells, we also determined their ability to induce T cell proliferation.

## 2 Material and methods

### 2.1 Bacterial strains and recombinant proteins

All pneumococcal strains used in this study are listed in **Table 1**. *S. pneumoniae* TIGR4Δ*cps*Δ*ply*, TIGR4Δ*cps*Δ*lgt*, D39Δ*cps* and D39Δ*cps*Δ*ply*Δ*lgt* mutant strains were described previously (26–29). D39Δ*cps*Δ*ply*, D39Δ*cps*Δ*ply*Δ*lgt* and TIGR4Δ*cps*Δ*ply*Δ*lgt* were generated accordingly using the construct pGEM-T EasyΔp*ly*::*cat* (26) in different genetic backgrounds [PN111 (27), PN220 (29), PN443 (28)].

**Table 1:** Pneumococcal strains used in this study.

Strain	Genotype	Resistance	Reference
PN421	TIGR4Δ*cps*Δ*ply*	Km^R^, Cm^R^	(26)
PN111	D39Δ*cps*	Km^R^	(27)
PN419	D39Δ*cps*Δ*ply*	Km^R^, Cm^R^	This work
PN443	TIGR4Δ*cps*Δ*lgt*	Km^R^, Erm^R^	(28)
PN821	TIGR4Δ*cps*Δ*ply*Δ*lgt*	Km^R^, Cm^R^, Erm^R^	This work
PN220	D39Δ*cps*Δ*lgt*	Km^R^, Erm^R^	(29)
PN773	D39Δ*cps*Δ*ply*Δ*lgt*	Km^R^, Cm^R^, Erm^R^	This work

Cm, chloramphenicol; Km, kanamycin; Erm, erythromycin.

For infection experiments, pneumococci were grown on blood agar plates (Oxoid) for 10 to 12 hours at 37°C and 5% CO_2_. The next day, pneumococci were cultured in Todd-Hewitt broth (Carl Roth) supplemented with 0.5% (w/v) yeast extract (Carl Roth) at 37°C to mid-log phase (optical density [OD] 600 nm, 0.35-0.45).

The generation of recombinant constructs and purification of lipidated DacB (LipDacB) and non-lipidated DacB or MetQ (sequence in **Supplementary Figure S1A**) were previously published (11, 15, 16). Recombinant lipidated MetQ (LipMetQ, sequence in **Supplementary Figure S1B**) was generated and purified as described (16). Briefly, the target gene *metQ* (sp_0149 in TIGR4; nt 70-852, aa 24-284) was amplified by PCR with the primer pair Pfor (5’-GCGCCATATGGGAAACTCAGAAAAGAAAGCAGA-3’) and Prev (5’-GCGCGGATCCTTAATGATGATGATGATGATGCCAAACTGGTTGATCC-3’) followed by insertion into the pETLip3 vector (contains the signal sequence of the outer surface protein A from *Borrelia burgdorferi*) using the restriction sites *Nde*I/*Bam*HI. The recombinant plasmid was transformed into *E. coli* ClearColi^®^ (Lucigen^®^), protein expression was induced with 1 mM isopropyl-β-d-1-thiogalactopyranoside, and purification of His_6_-tagged LipMetQ from the supernatant of the ClearColi^®^ lysate was performed by affinity chromatography using a HisTrap™ Ni-NTA column in the ÄKTApurifier (GE Healthcare). For better solubilization of the lipidated protein, 20 mM zwitterionic detergent CHAPS [3-((3-cholamidopropyl)dimethylammonio)-1-propanesulfonate] was added to the lysate. The purified protein was dialyzed against phosphate-buffered saline (PBS) and analyzed for purity by SDS-PAGE and immunoblotting. Recombinant lipidated MetQ was analyzed in a top-down strategy using liquid chromatography–mass spectrometry (LC–MS) as previously described (16). Lipidation of MetQ was confirmed (**Supplementary Figures S2**, **S3**) and detailed interpretation is provided in the **Supplementary Materials**.

### 2.2 Isolation of PBMCs or human monocytes and differentiation to moDCs and MDMs

PBMCs were isolated from buffy coats via Lymphoprep density gradient centrifugation (Stemcell Technologies). After several washes and a step of red blood cell lysis, PBMCs were either used directly or stored in FCS containing 10% (v/v) DMSO at -170°C.

Human monocytes were isolated from buffy coats or PBMCs via CD14 S-pluriBead anti-human beads (PluriSelect) according to manufacturer’s instructions. The moDCs were generated by culturing monocytes for 5 days in RPMI1640 medium (Cytiva) supplemented with 10% (v/v) heat inactivated fetal calf serum (FCS; Sigma-Aldrich), 89 ng/ml GM-CSF and 22 ng/ml IL-4 (both ImmunoTools) with a change of medium on day 3. Differentiation to MDMs was achieved by culturing monocytes for 5 days in RPMI1640 medium supplemented with 10% (v/v) heat inactivated FCS and 25 ng/ml GM-CSF. The medium was exchanged on the third day.

### 2.3 Infection and stimulation of moDCs and MDMs

Pneumococcal infections and protein stimulations were performed in RPMI1640 medium supplemented with 10% (v/v) heat inactivated FCS in 96-well plates. For pneumococcal infection, 1 × 10^5^ moDCs were infected with different pneumococcal strains at a multiplicity of infection (MOI) of 10. After 2 h, the medium was removed and extracellular bacteria were killed by addition of medium containing antibiotics (100 µg/ml gentamicin (Sigma-Aldrich), 100 µg/ml streptomycin/100 IU/ml penicillin G (Hyclone)). After a total of 24 h, the moDCs were prepared for flow cytometry.

For protein stimulation, 1 × 10^5^ moDCs or MDMs were stimulated with 1 µg DacB, MetQ or their lipidated versions LipDacB or LipMetQ, respectively. To warrant comparable conditions, the medium was supplemented with the same antibiotics cocktail as mentioned above. After 24 h, moDCs or MDMs were prepared for flow cytometry.

In all infection and stimulation assays, unstimulated cells cultured in medium were used as negative control, whereas cells incubated with 100 ng/ml LPS (Sigma-Aldrich), 200 ng/ml Pam2CSK4, or 200 ng/ml Pam3CSK4 (both InvivoGen), were used as positive controls.

### 2.4 Tag-it Violet^™^ T cell proliferation assay

PBMCs (1 × 10^7^) were labeled with 5 µM Tag-it Violet^™^ proliferation dye (BioLegend) according to manufacturer’s instructions. Tag-it Violet^™^-labeled cells were seeded at a density of 1 × 10^6^ cells/well in a 24-well plate in AIM-V™ medium (Thermo Fisher Scientific) supplemented with 2% (v/v) heat inactivated human AB serum (Sigma-Aldrich). Cells were stimulated with 1 µg/ml of pneumococcal proteins DacB, LipDacB, MetQ, LipMetQ, or 200 ng/ml Pam3CSK4, respectively, in replicate wells per condition at 37°C and 5% CO_2_ for 5 and 7 days. Afterwards, cells were harvested, the replicates pooled and prepared for flow cytometry.

### 2.5 Flow cytometry

For flow cytometric analyses of moDCs and MDMs, cells were labeled using the Zombie Aqua™ Fixable Viability Kit (BioLegend) to stain dead cells. Human TruStain FcX™ (BioLegend) was used according to the manufacturer’s instructions to block unspecific binding of immunoglobulins to Fc receptors on cells. Cells were incubated with fluorochrome-labeled monoclonal antibodies for 30 min at 4°C in the dark and washed between each working step. The following antibodies were used to stain moDCs: cluster of differentiation (CD) 209 (PE, Clone DCN47.5, Miltenyi Biotec), CD40 (BV421, Clone 5C3, BioLegend), CD80 (APC, Clone 2D10, BioLegend), CD83 (BV711, Clone HB15e, BioLegend), CD86 (PE-Cy7, Clone BU63 BioLegend), and major histocompatibility complex (MHC) II (FITC, Clone L243, BioLegend). For staining of MDMs, in addition to the CD80 and CD86 antibodies, MHCII (BV421, Clone L243, BioLegend) and CD68 (PE, Clone Y1/82A, BioLegend) were used.

For the T cell proliferation assay, Tag-it Violet^™^ labeled cells were harvested, washed and stained with Zombie NIR™ Fixable Viability Kit according to the manufacturer’s instructions to exclude dead cells. Next, cells were washed and stained for 30 min at 4°C in the dark with the following fluorochrome-labeled antibodies: CD3 (APC, Clone UCHT1, BioLegend), CD4 (FITC, Clone RPA-T4, BioLegend) and CD8a (PE, CloneRPA-T8, BioLegend). The respective gating strategies are shown in **Supplementary Figure S4**. Data were acquired using a FACSAria™ III flow cytometer and FACSDiva™ software 8.0 (both BD Biosciences) and analyzed using FCS Express 7 software (*De Novo* Software).

### 2.6 Cytokine measurements

Cytokine concentrations were determined in supernatants of infected or stimulated moDCs and MDMs using the LEGENDplex™ human inflammation panel (13-plex) kit (BioLegend) according to the manufacturer’s instructions. Data were acquired with a FACSAria™ III flow cytometer using FACSDiva™ software 8.0 (both BD Bioscience) and analyzed using LEGENDplex™ software (BioLegend).

### 2.7 Statistics

Statistical analysis was performed using GraphPad Prism 8 software (La Jolla, CA, United States), with significant differences determined by the Kruskal Wallis test in combination with Dunn’s test of multiple comparisons. A *p*-value less than 0.05 was considered statistically significant.

## 3 Results

### 3.1 Deletion of *lgt* does not differentially affect DC maturation at the whole-cell level

The effect of pneumococcal lipoproteins on DC maturation at the whole-cell level was investigated by infecting moDCs with pneumococcal strains deficient in Lgt from two different serotypes and analyzing the expression or expansion of common DC activation markers. Absence of Lgt leads to the expression of nonlipidated lipoproteins which disappear from the surface proteome but accumulate in the exoproteome (29). In addition, all pneumococcal strains used were nonencapsulated and deficient in pneumolysin (Ply), as Ply has previously been shown to impair the maturation of DCs (30, 31).

**Figure 1:**
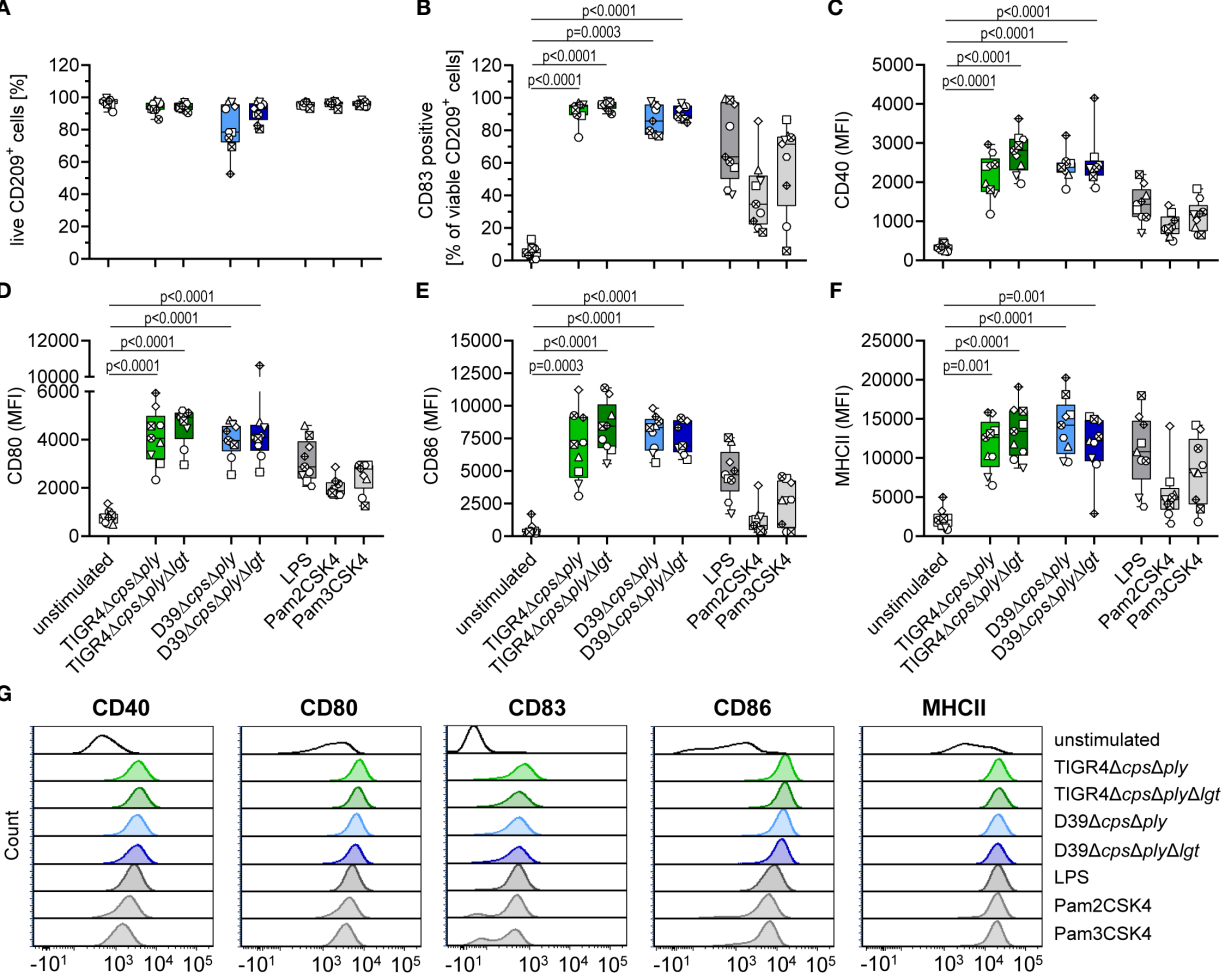
Infection of human moDCs with pneumococcal strains induces maturation. Human moDCs were infected with indicated pneumococcal strains (MOI 10). Positive controls included stimulation with LPS (100 ng/ml), Pam2CSK4 (200 ng/ml), or Pam3CSK4 (200 ng/ml). MoDCs viability **(A)** and phenotype **(B–F)** were determined by flow cytometry (n=9). Representative histograms for each surface marker are shown in **(G)**. The maturation process was evaluated based on the expression of CD40 **(C)**, CD80 **(D)**, CD86 **(E)** and MHCII **(F)** as well as the frequency of CD83^+^ cells **(B)**. The data in **(A–F)** are displayed as box plots. Each dot represents the response of one donor. The level of significance was determined using Kruskal-Wallis test with Dunn`s post-test. (MFI, mean fluorescence intensity).

Viability and maturation of moDCs was assessed via flow cytometry based on Live/Dead staining and the expression of the activation markers CD40, CD80, CD86 and MHC II as well as the expansion of CD83^+^ cells (**Figure 1**). It was observed that independent of serotype or *lgt* deletion, the majority of the moDCs remained viable (**Figure 1A**) and showed a clear maturation process (**Figures 1B–G**). The expression of all measured receptors was significantly increased in response to pneumococcal infections and comparable or even higher than our included positive controls. However, we did not detect differential effects on moDC maturation depending on the presence of Lgt at the whole-cell level. This suggests that other pneumococcal factors have the potential to influence DC maturation, thereby overriding the effects of *lgt* deletion.

### 3.2 Lipidation of pneumococcal proteins triggers moDC maturation and activation

**Figure 2:**
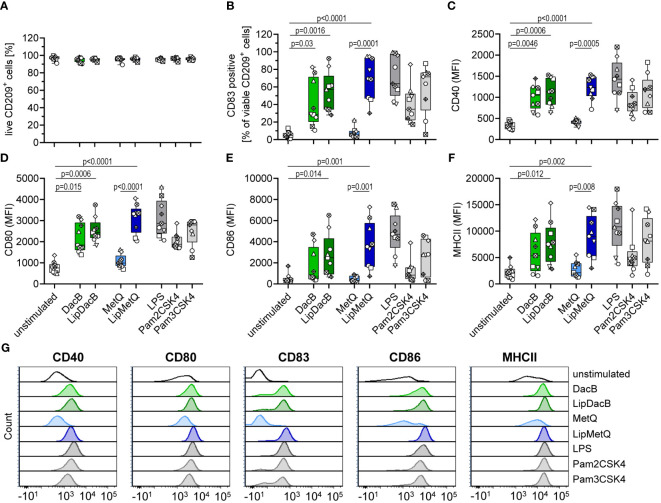
Lipidation increases activation potential of pneumococcal proteins in moDC stimulations. Human moDCs were stimulated with the pneumococcal proteins DacB and MetQ, both in a lipidated and non-lipidated version (1.0 µg). Positive controls included stimulation with LPS (100 ng/ml), Pam2CSK4 (200 ng/ml), or Pam3CSK4 (200 ng/ml). MoDCs viability **(A)** and phenotype **(B–F)** were determined by flow cytometry (n=9). Representative histograms for each surface marker are shown in **(G)**. The maturation process was evaluated based on the expression of CD40 **(C)**, CD80 **(D)**, CD86 **(E)** and MHCII **(F)** as well as the frequency of CD83^+^ cells **(B)**. The data in **(A–F)** are displayed as box plots. Each dot represents the response of one donor. The level of significance was determined using Kruskal-Wallis test with Dunn`s post-test. (MFI, mean fluorescence intensity).

Because there were no differences in moDC maturation at the whole-cell level that correlated with Lgt expression, we investigated the impact of lipidation on two individual pneumococcal lipoproteins, namely DacB and MetQ, which have been identified as highly conserved and potential vaccine candidates in mouse models (15, 16). MoDCs were stimulated with the lipidated and nonlipidated version of the proteins followed by the assessment of viability and maturation process (**Figure 2**). The viability of moDCs was not affected by either non-lipidated or lipidated versions of DacB or MetQ (**Figure 2A**). However, lipidation of MetQ played a crucial role in dendritic cell activation as LipMetQ induced a strong moDC maturation process, whereas stimulation with non-lipidated MetQ was similar to the unstimulated control (**Figures 2B–G**). Remarkably, the non-lipidated version of DacB was already able to activate moDCs, suggesting that lipidation is not necessarily required and DacB has an intrinsic capacity to activate DCs. Nonetheless, lipidation of DacB led to a slight increase in maturation potential given that CD86 and MHCII were significantly upregulated only after stimulation with LipDacB compared with the unstimulated control (**Figures 2E, F**). In conclusion, with the exception of MetQ, all pneumococcal lipoproteins used were able to activate human primary moDCs, with lipidation enhancing the activation potential.

### 3.3 Pneumococcal lipoproteins induce strong cytokine release in moDC stimulations

**Figure 3:**
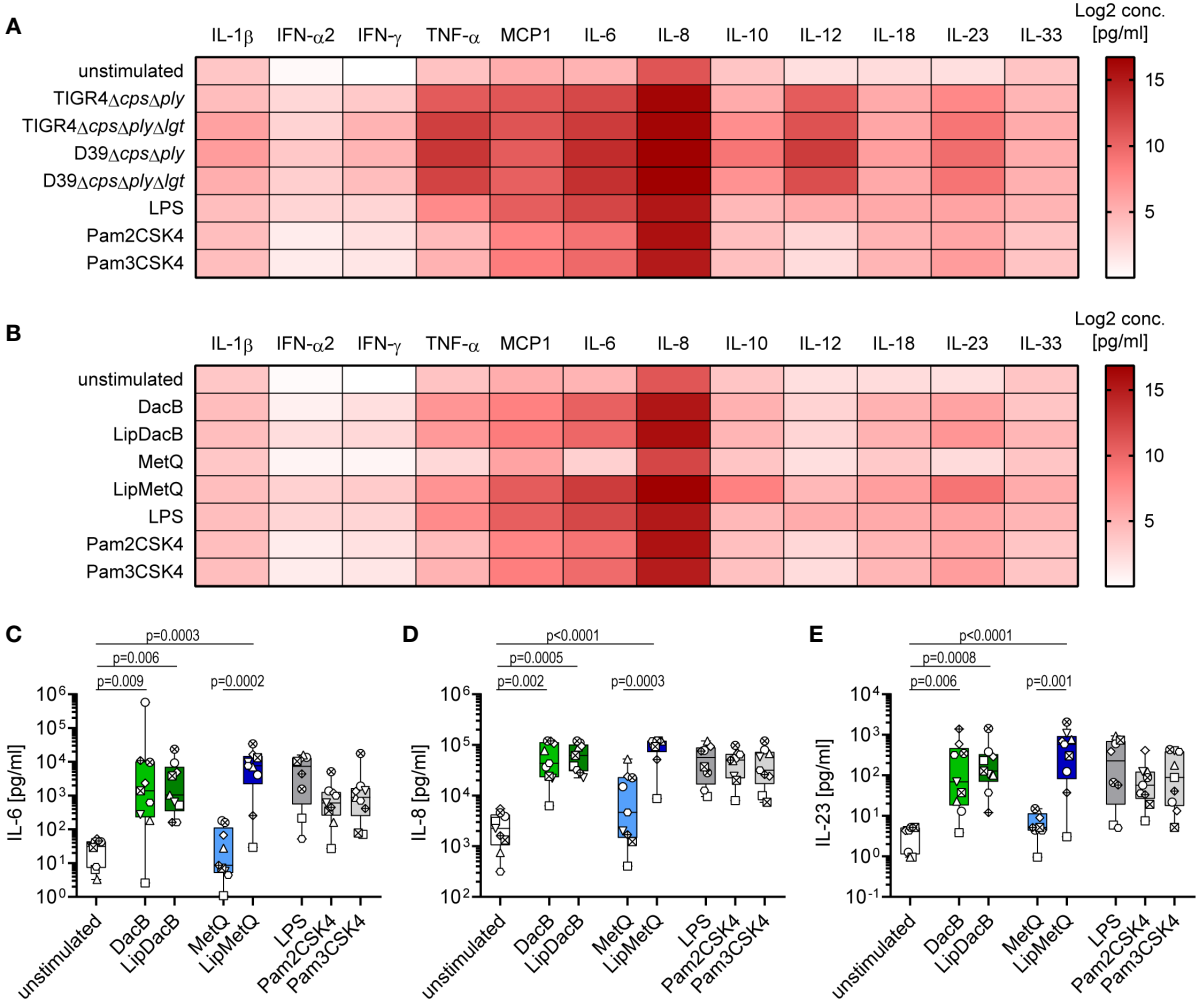
Cytokine responses of moDCs after pneumococcal infection or stimulation with pneumococcal lipoproteins. Cytokine secretion of infected **(A)** or protein-stimulated **(B)** human moDCs was determined using the LEGENDplex™ human inflammation panel in flow cytometry (n=9). The heat maps represent the Log2 concentrations of measured cytokines in supernatants of moDCs in pg/ml. Original data are displayed in **Supplementary Figure S6** and **(C–E)**. The concentrations of IL-6 **(C)**, IL-8 **(D)** and IL-23 **(E)** were measured in supernatants of moDCs stimulated with lipidated or non-lipidated proteins. The data are displayed as box plots. Each dot represents the response of one donor. The level of significance was determined using Kruskal-Wallis test with Dunn`s post-test.

Next, we examined the cytokine release of moDCs induced by pneumococcal strains and lipoproteins, respectively, as cytokines play a critical role in shaping the adaptive immune response (24). Consistent with the previous findings, all tested pneumococcal strains induced a robust cytokine response irrespective of serotype or *lgt* mutation (**Figure 3A**, **Supplementary Figure S5**). Especially the release of MCP1, IL-6, IL-8 and IL-12p70 was highly increased (**Figure 3A**, **Supplementary Figures S5E, F, G, I**). A similar pattern to the previous results was also measured when moDCs were incubated with our proteins. Stimulation with MetQ was comparable to the cytokine concentrations of the unstimulated control (**Figure 3B**, **Supplementary Figure S6**). In contrast, stimulation with DacB, LipDacB and LipMetQ resulted in a strong cytokine response e.g., for IL-8 (**Figure 3D**). Because it was shown that IL-17 plays an important role in protection against pneumococcal colonization in mice (15), we were especially interested in cytokines involved in triggering T helper (Th) 17 cell responses including IL-6 and IL-23 (32, 33). Interestingly, the levels of both cytokines were significantly increased when moDCs were stimulated with DacB, LipDacB and LipMetQ (**Figures 3C, E**).

### 3.4 The effects of lipidated and non-lipidated pneumococcal proteins are not DC-specific

**Figure 4:**
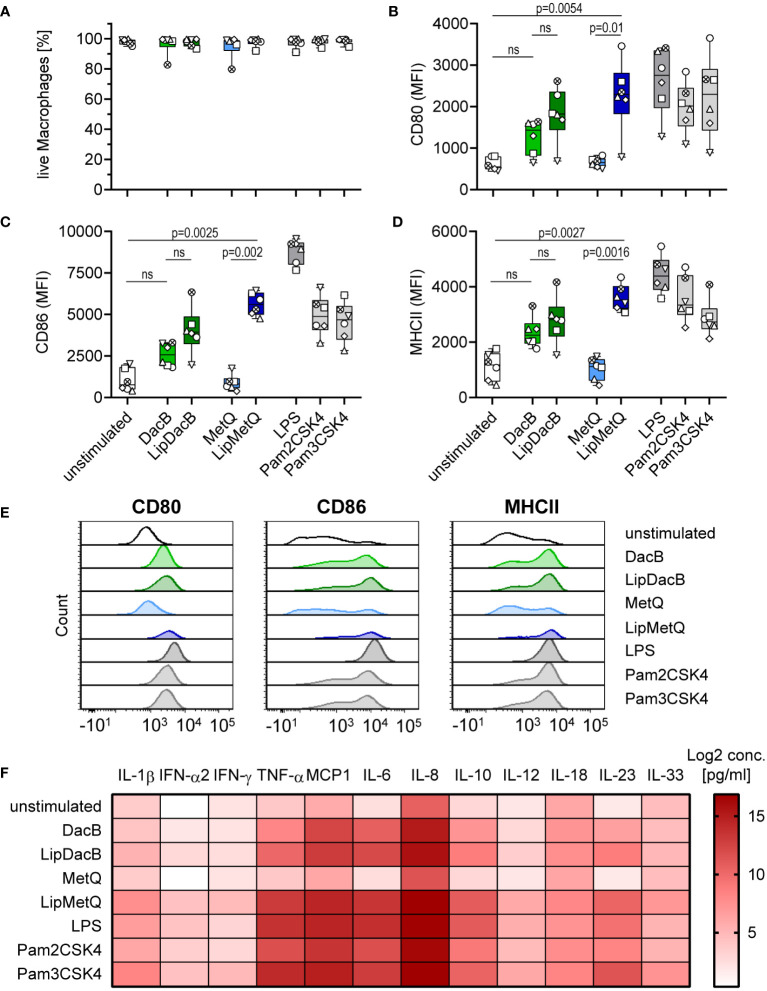
Lipidation-mediated activation is reproducible in human MDMs. Human MDMs were stimulated with the pneumococcal proteins DacB and MetQ, both in a lipidated and non-lipidated version (1.0 µg). Positive controls included stimulation with LPS (100 ng/ml), Pam2CSK4 (200 ng/ml), or Pam3CSK4 (200 ng/ml). MDMs viability **(A)** and phenotype **(B–D)** were determined by flow cytometry (n=6). Representative histograms for each surface marker are shown in **(E)**. The activation process was evaluated based on the expression of CD80 **(B)**, CD86 **(C)** and MHCII **(D)**. The data in **(A–D)** are displayed as box plots. Each dot represents the response of one donor. The heat map **(F)** represents the Log2 concentrations of measured cytokines in supernatants of MDMs (in pg/ml) stimulated with lipidated or non-lipidated proteins. Original data are displayed in **Supplementary Figure S7**. The level of significance was determined using Kruskal-Wallis test with Dunn`s post-test. (MFI, mean fluorescence intensity; ns, not significant).

The results showed that for MetQ lipidation was necessary to induce maturation of moDCs, whereas non-lipidated DacB itself already exhibited an activation potential. Next, we elucidated whether these effects are DC-specific or can be reproduced with other APCs. Therefore, MDMs were stimulated with lipidated and non-lipidated pneumococcal proteins and analyzed for activation and cytokine release by flow cytometry (**Figure 4**). The activation of MDMs was evaluated by assessing the expression of CD80, CD86 and MHCII. The proteins had no cytotoxic effects on MDMs (**Figure 4A**). In line with the moDC results, DacB, LipDacB and LipMetQ showed activation of MDMs and increased expression of the investigated markers (**Figures 4B–E**), although this was only statistically significant for LipMetQ. In contrast, MDMs stimulated with non-lipidated MetQ exhibited receptor expression comparable to the unstimulated control. Furthermore, activation was tested by analyzing secreted cytokines in supernatants of stimulated MDMs (**Figure 4F**, **Supplementary Figure S7**). In contrast to MetQ, which did not stimulate cytokine release, the levels of most of the cytokines was increased after MDMs were stimulated with DacB, LipDacB and LipMetQ. We observed that protein lipidation significantly upregulated IL-6, IL-10 and IL-23 cytokine release (**Figure 4F**, **Supplementary Figures S7F, H, K**). These results demonstrate that the activation potential of pneumococcal lipoproteins is not limited to a specific type of APCs. Instead, they elicit a robust innate immune response in general.

### 3.5 Lipidation triggers initiation of an adaptive immune response

**Figure 5:**
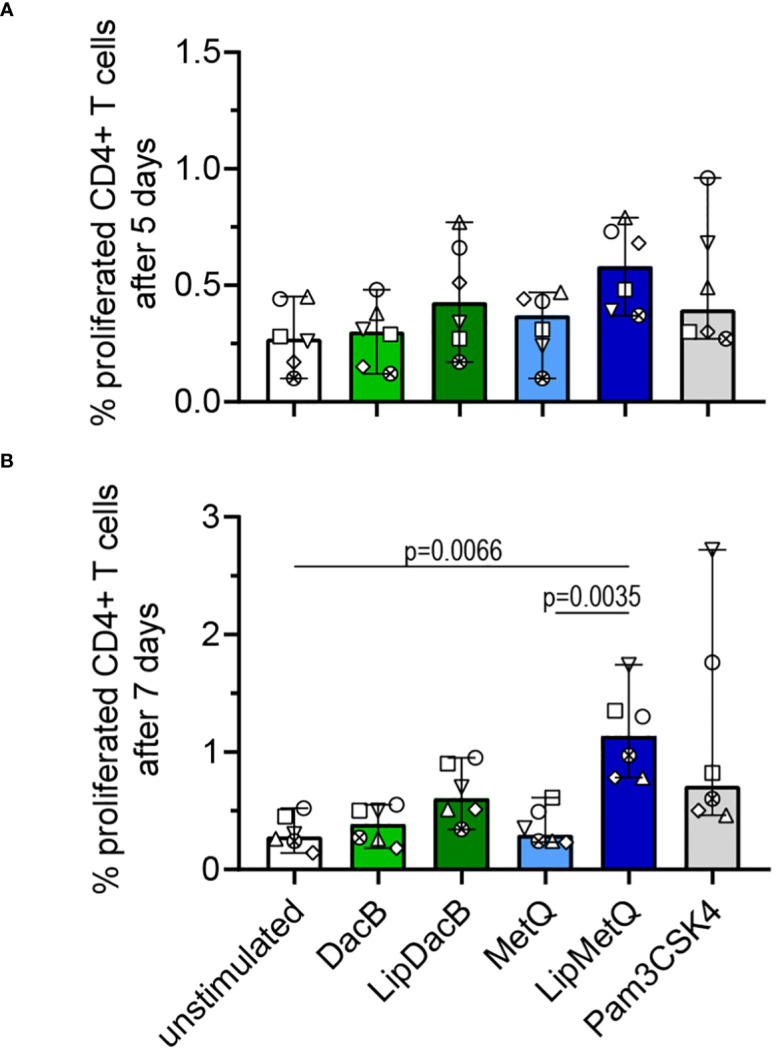
Lipidated proteins induce proliferation of CD4^+^ T cells. Human PBMCs were stimulated with the pneumococcal proteins DacB and MetQ, both in a lipidated and non-lipidated version (1.0 µg). As a control, stimulation with Pam3CSK4 (200 ng/ml) was included. Proliferation of CD3^+^CD4^*^ T cells was analyzed by flow cytometry (n=6) after five **(A)** or seven **(B)** days of stimulation. The data are displayed as columns with dots representing the response of one donor. The level of significance was determined using Kruskal-Wallis test with Dunn`s post-test.

In order to achieve comprehensive vaccine protection, it is important to induce a cellular immune response. To address this, we investigated whether pneumococcal lipoproteins are able to initiate T cell proliferation and assessed the role of lipidation in this process. Therefore, tag-it Violet^™^ labeled human PBMCs were stimulated with lipidated and non-lipidated proteins followed by assessment of CD3^+^CD4^+^ T cell proliferation after 5 or 7 days by flow cytometry (**Figure 5**). After 5 days, an increased number of proliferated T cells was already detected for both lipidated protein versions (**Figure 5A**). For LipMetQ, the effect became statistically significant after two further days of stimulation. (**Figure 5B**). Stimulation with the non-lipidated proteins displayed the same pattern as the unstimulated control. Overall, these data indicate that lipidation of pneumococcal lipoproteins leads to an increased ability to induce CD4^+^ T cell proliferation and thus trigger an adaptive immune response.

## 4 Discussion

Ongoing research aims to devise novel vaccine strategies against pneumococcal infections, addressing limitations of currently available vaccines such as inadequate serotype coverage and high manufacturing costs. Pneumococcal lipoproteins are gaining attention as promising vaccine candidates, given their surface exposure, conservation across serotypes, and high immunogenicity through recognition by human APCs via TLR2. However, only a few studies so far have examined the potential of lipoproteins as vaccine candidates in a human background and there are insufficient data on the impact of lipidation. In the present study, we show that pneumococcal lipoproteins are able to induce maturation of human moDCs. Lipidation of recombinant proteins increased their activation potential, an effect that was not DC-specific and reproducible with primary macrophages. Furthermore, lipidation proved to be essential for stimulation and proliferation of human CD4+ T cells.

DCs play a crucial role in initiating an effective immune response against bacterial infections. The significance of DCs as key contributors to an efficient immune response in pneumococcal infection was previously shown. Using two mouse models, it was demonstrated that expansion of DCs in the nasal cavity is important for antibody responses after colonization with pneumococci and subsequent protective immunity against invasive pneumococcal disease (34). However, several studies have shown that activation of DCs by pneumococci is impaired by the pore forming toxin Ply (30, 31, 35). For this reason, we generally used pneumococcal mutant strains deficient for Ply in our study. Pneumococcal lipoproteins are translated in the cytoplasm and released as preprolipoproteins. Afterwards, Lgt is responsible for the covalent binding of the lipoprotein precursor to the cytoplasmic membrane. In a next step, the signal peptide is cleaved by the lipoprotein signal peptidase II (Lsp) resulting in a mature lipoprotein (12). Earlier studies showed that in the absence of Lgt the attachment of lipoproteins to the bacterial surface is drastically reduced and that these lipoproteins lack lipidation (29). Therefore, we infected moDCs with Lgt deficient pneumococcal strains to investigate the impact of lipoproteins on DC activation at the whole-cell level.

We observed that irrespective of the absence of Lgt, pneumococci activated human moDCs both at the receptor and cytokine level. Initially, this discovery appeared contradictory to existing literature, as numerous studies have indicated that an effective immune response against pneumococci may rely on the presence of pneumococcal lipoproteins (19, 21). We hypothesize that any lipoprotein-based effects are likely to be masked by other pneumococcal virulence factors that can induce DC maturation (36). This hypothesis is supported by a study examining the effects of pneumococcal extracellular vesicles (EVs) on moDCs. EVs contain choline-binding as well as sortase-anchored proteins and were shown to be able to induce maturation of dendritic cells (37). Furthermore, another study clearly shows the activation potential of the specific choline-binding protein PspC (also referred to as CbpA) on human DCs (38). As the pneumococcal surface is perfectly equipped with proteins from these families, this could be the reason for the observed induction of DC maturation even if no lipoproteins are incorporated into the membrane.

To advance our investigations on the impact of pneumococcal lipoproteins, we decided to focus on specific representatives, namely DacB and MetQ, in both lipidated (LipDacB and LipMetQ) and non-lipidated (DacB and MetQ) versions. The great potential of the lipid moiety as part of a vaccine formulation has already been demonstrated in mouse models, as lipidation status plays a key role in protection against pneumococci (15–17, 20). To validate this observation in a human context, we stimulated moDCs with the specific lipoproteins. Both proteins have already been shown to contribute to pneumococcal diseases, with MetQ involved in severe pulmonary infections (12, 39, 40), and DacB impacting adherence, colonization, and antibiotic resistance of pneumococci (11, 41, 42).

While lipidation of MetQ resulted in a significantly increased immunostimulating capacity, we observed that DacB, even without lipidation, could induce maturation of human moDCs. This was especially shown by expansion of CD83 positive cells and the increased expression of CD40 and CD80. Lipidation of DacB only marginally enhanced its activation potential. Nevertheless, our findings suggest that the lipid moiety of MetQ is crucial for immune recognition, in contrast to DacB, underscoring the substantial impact of the lipid status of vaccine candidates and the notable immunogenic potential of DacB itself. Further investigations are needed to determine whether this is related to the enzymatic l,d-carboxypeptidase activity of DacB (11). Importantly, this finding was not restricted to DCs, as we observed a similar effect upon stimulation of human MDMs. Following stimulation with DacB, LipDacB, and LipMetQ, human macrophages exhibited a clear proinflammatory phenotype, with an additional increase for the lipidated protein versions. Overall, we were able to demonstrate the activation potential of pneumococcal lipoproteins on human APCs at both receptor and general cytokine level – a pivotal step in the exploration of lipoproteins as novel vaccine candidates and the potential adjuvant properties of lipidation. These results align with other studies indicating that protein immunogenicity can be enhanced by lipidation (16, 20).

Earlier studies in mice demonstrated that protection against pneumococcal colonization was closely correlated with elevated levels of IL-17 (15, 16, 43–46), primarily produced by Th17 cells. Moreover, in human skin models treated with *S. pneumoniae*, the crucial role of Th17 cells was highlighted (47). A recent study revealed that human tissue showed enhanced titers of IL-17 and IL-22 in response to pneumococcal infection. In line with that, the recruited T cells were primarily Th17 cells (47). By the release of IL-6 and IL-23, APCs are able to shape the differentiation of receptor-activated CD4^+^ T cells into Th17 cells (24, 32, 33). Additionally, it has been shown that the level of IL-17 positively corelates with the expression of CD40 on human DCs (48). In our study, we determined enhanced levels of IL-6 and IL-23 in supernatants of DCs stimulated with DacB, LipDacB und LipMetQ, accompanied by an increased expression of CD40. Once again, the highest titers were observed following stimulation with lipidated proteins. In the supernatants of macrophages, the results became even more pronounced. For both lipidated proteins we observed strongly increased levels of IL-6 and IL-23. This effect could be demonstrated in particular by stimulation with individual proteins, as *ply* expressing pneumococcal strains are able to suppress the production of IL-6 via Ply at the whole-cell level (49). However, these findings on the protein level provide a robust basis for further vaccine-oriented research. It is commonly known that both receptor interactions and cytokine response are required for a comprehensive T cell response, enabling a cellular immune response essential for complete vaccine-based protection. This is underscored by several studies showing that protection against pneumococci in mice relies on CD4^+^ T cells and the release of IL-17 (50, 51).

Previous research has established the capability of pneumococcal lipoproteins to trigger a T cell-mediated immune response (52). However, this study did not focus on DacB and MetQ and the investigation of the impact of lipidation was missing. Here, we have now demonstrated that lipidation of pneumococcal proteins enhances their ability to induce an adaptive immune response as evidenced by a T cell proliferation assay. Although stimulation with LipDacB did not show a significant increase in proliferation, there is a noticeable time-related trend. Interestingly, stimulation with LipMetQ resulted in a significant proliferation of CD4^+^ T cells after 7 days. Of note, despite the seemingly small percentages of proliferation, they are comparable to values obtained from T cell proliferation experiments reported in previous studies (52).

In conclusion, our study underscores the potential of lipoproteins as promising candidates for the development of novel vaccine strategies. This aligns with findings from various other studies investigating the use of lipoproteins as vaccine candidates against other bacteria and viruses (53–55). However, most of these studies have been conducted in mice and lack human data. Here, we have demonstrated the potential of pneumococcal lipoproteins as novel vaccine candidates, emphasizing their enhanced immunogenicity after attachment of a lipid moiety in a human context. The lipidated versions of DacB and MetQ exhibited robust activation of human primary APCs and further induced the proliferation of human CD4^+^ T cells. Future investigations should delve into the activation potential of non-lipidated DacB and further elucidate the potential of lipidation as adjuvantation for pneumococcal vaccine strategies.

## Data availability statement

The raw data supporting the conclusions of this article will be made available by the authors, without undue reservation.

## Ethics statement

Buffy coats obtained from healthy blood donors were anonymously provided by the blood bank at the University Medicine Greifswald. All experiments were approved by the Ethical Research Committee at the University Medicine Greifswald (Ref. No. BB 014/14). All experiments were carried out in accordance with the approved guidelines.

## Author contributions

AP: Data curation, Investigation, Methodology, Writing – original draft. DS: Investigation, Methodology, Writing – review & editing. SH: Formal analysis, Supervision, Writing – review & editing, Conceptualization, Funding acquisition, Project administration, Resources, Validation. FV: Data curation, Formal analysis, Investigation, Methodology, Supervision, Writing – original draft, Writing – review & editing.
