# Supplementary material for: Lipidation of pneumococcal proteins enables activation of human antigen-presenting cells and initiation of an adaptive immune response
Source: Front Immunol. 2024 Apr 22;15:1392316. doi: 10.3389/fimmu.2024.1392316 (PMC11070533; doi:10.3389/fimmu.2024.1392316)
Supplement: Supplementary file 1 [file DataSheet_1.pdf]

Supplementary Material

Lipidation of pneumococcal proteins enables activation of human antigen-presenting cells and initiation of an adaptive immune response

Antje D. Paulikat, Dominik Schwudke, Sven Hammerschmidt*, Franziska Voß

*** Correspondence:** Sven Hammerschmidt, sven.hammerschmidt@uni-greifswald.de

1 Supplementary Figures

A MetQ

```
GSSHHHHHHMSGENLYFQGASGNSEKKADNATTIKIATVNRSGSEEKRWDKIQELVKKDG
ITLEFTEFTDYSQPNKATADGEVDLNAFQHYNFLNNWNKENGKDLVAIADTYISPIRLYS
GLNGSANKYTKVEDIPANGEIAVPNDATNESRALYLLQSAGLIKLDVSGTALATVANIKE
NPKNLKITELDASQTARSLSSVDAAVVNNTFVTEAKLDYKKSLFKEQADENSKQWYNIIV
AKKDWETSPKADAIKKVIAAYHTDDVKKVIEESSDGLDQPVW
```

B LipMetQ

```
MKIKKWLGLAALATVAGLALAA|CKQNVSSHMGNSEKKADNATTIKIATVNRSGSEEKRW
DKIQELVKKDGITLEFTEFTDYSQPNKATADGEVDLNAFQHYNFLNNWNKENGKDLVAIA
DTYISPIRLYSGLNGSANKYTKVEDIPANGEIAVPNDATNESRALYLLQSAGLIKLDVSG
TALATVANIKENPKNLKITELDASQTARSLSSVDAAVVNNTFVTEAKLDYKKSLFKEQAD
ENSKQWYNIIVAKKDWETSPKADAIKKVIAAYHTDDVKKVIEESSDGLDQPVWHHHHHH
```

Supplementary Figure 1. Protein sequences of recombinant lipidated and non-lipidated MetQ. (A) For non-lipidated MetQ, additional amino acids derived from the pTP1 expression vector are underlined followed by the protein sequence for MetQ. (B) For lipidated LipMetQ, OspA signal peptide that is cleaved off after posttranslational modification is indicated by italic letters. Additional amino acids derived from OspA are underlined followed by the protein sequence for MetQ. The cysteine (bold letter) is the site of lipidation. (A, B) The C-terminal His_6-tags are highlighted by red letters.

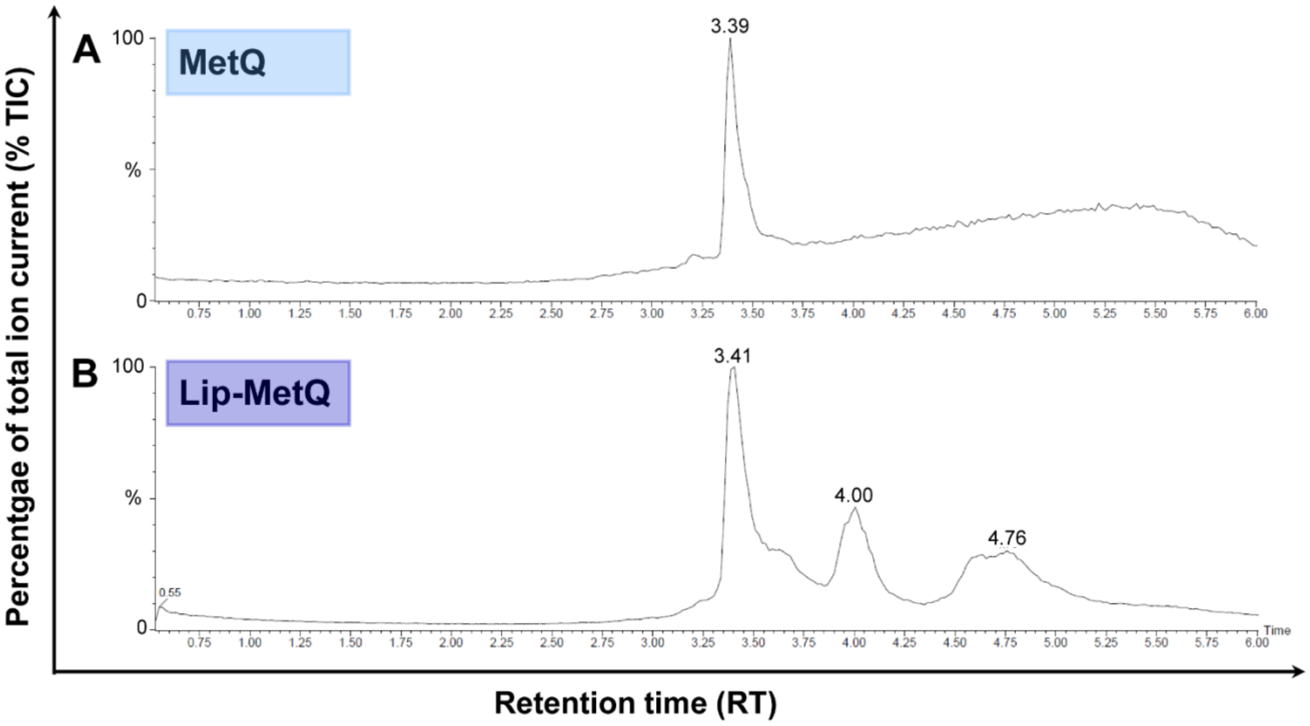

Supplementary Figure 2. LC-MS analysis of recombinant lipidated and non-lipidated MetQ. MetQ (A) and LipMetQ (B) were analyzed by LC-MS. Separation of MetQ, LipMetQ and associated lipids was enabled by earlier described LC conditions[1]. The total ion count is represented as chromatographic traces. The peaks at retention time (RT) 3.4 min in both panels result from non-lipidated MetQ, respectively. Only in panel B) LipMetQ is detectable at RT 4.0 min. Furthermore, at RT 4.76 min phospholipids from cell membranes were identified. Mass spectra of the chromatographic peaks are shown in Fig. S3.

[1] Voß F, van Beek LF, Schwudke D, Ederveen THA, van Opzeeland FJ, Thalheim D et al. Lipidation of Pneumococcal Antigens Leads to Improved Immunogenicity and Protection. Vaccines (Basel) 2020; 8(2).

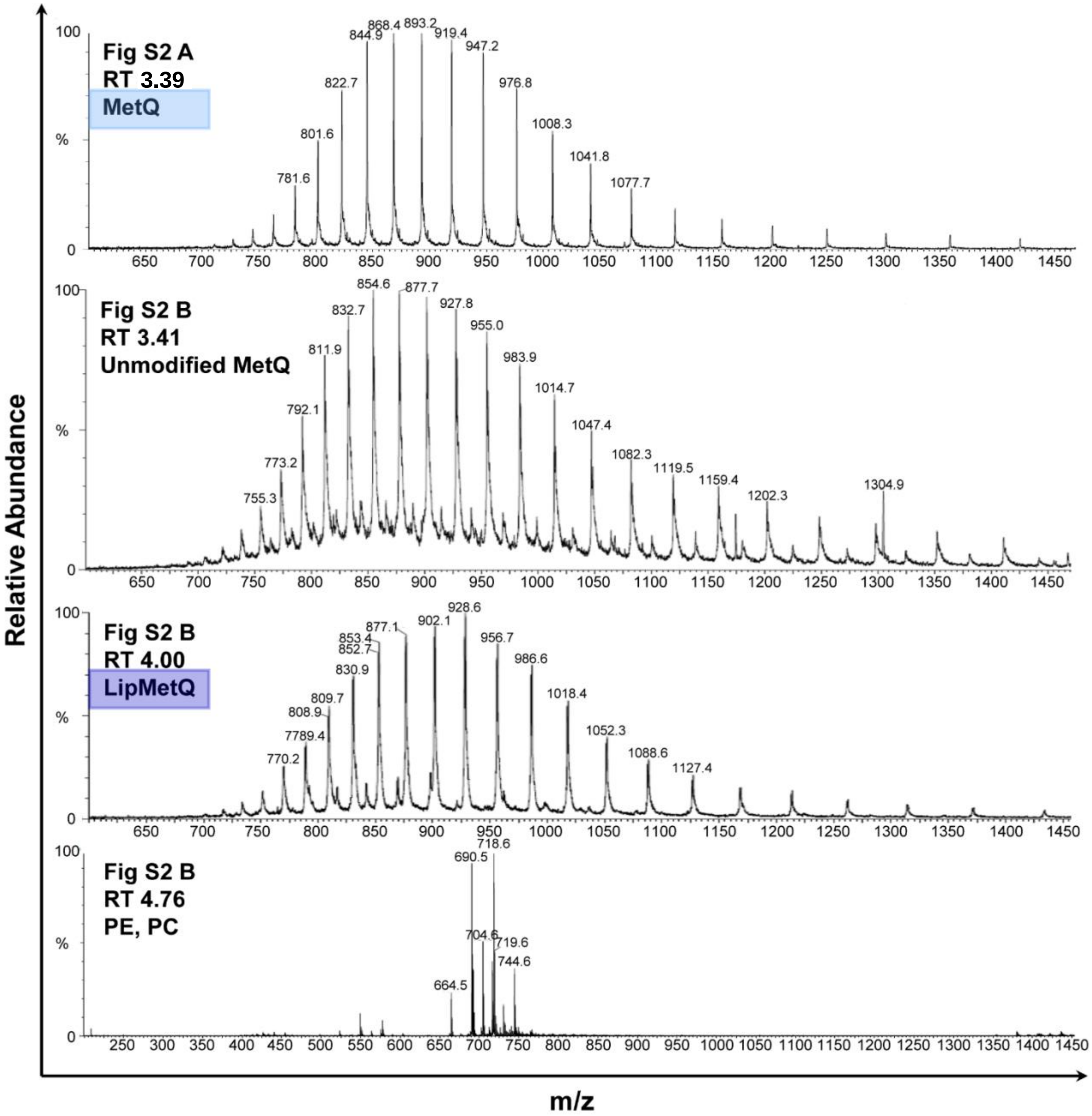

Supplementary Figure 3. Electrospray mass spectrometry of recombinant lipidated and non-lipidated MetQ. MetQ and LipMetQ were analyzed by LC-MS. Fig. S2A) For MetQ, a mass of 31,225 ± 1.2 Da was measured at RT 3.39 min (Fig. S2A), which is close to the expected mass of 31,221 Da for the recombinant MetQ (Fig. S1A). Fig. S2B) HPLC elution profiles of LipMetQ were composed of three major components: i) posttranslationally unmodified protein (RT: 3.41 min), ii) lipidated protein (RT: 4.00 min), and iii) phospholipids (RT: 4.76 min). Two protein species were identified at RT 3.41 min with 32,436 Da / 32,467 Da representing posttranslationally unmodified protein still carrying the OspA signal sequence (Fig. S1B). The averaged spectrum for the peak RT 4.00 min revealed two proteins with a mass of 31,511 Da and 31,539 Da. The expected lipidated form, in which three palmitate residues are attached to the N-terminal cysteine of the protein sequence, would have a mass of 31,508 Da. The protein form with a mass difference of 28 Da indicates an elongated chain with two CH_2 units, likely stearate. The signal detected at RT 4.76 min

corresponds to major phospholipids phosphatidylethanolamine (PE) and phosphatidylglycerol (PG) derived from bacterial membrane.

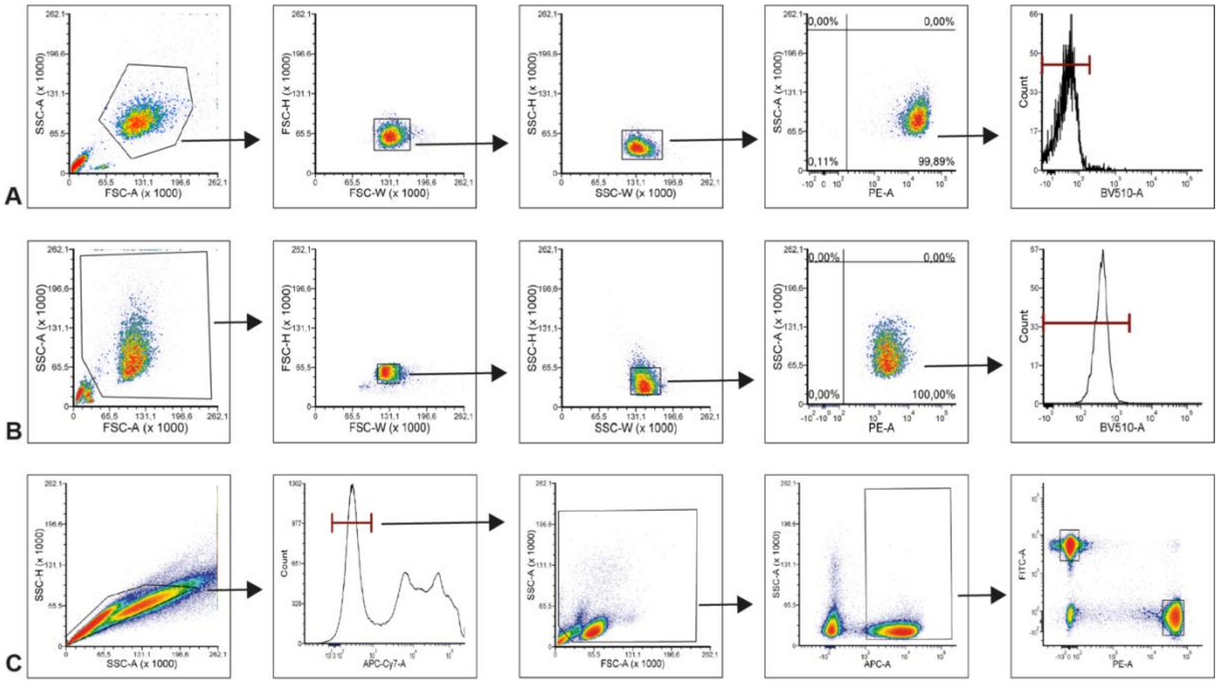

Supplementary Figure 4. Gating strategy for flow cytometry.Gating strategy for identification of human moDCs (A), human MDMs (B) and CD4^{+} T cells (C). Doublets were excluded by consecutive gating of FSC-H/FSC-W and SSC-H/SSC-W. MoDCs (A) and MDMs (B) were identified based on the expression of cell type-specific markers CD209-PE (A) and CD68-PE (B), respectively. Dead cells were excluded by using the Zombie Aqua™ Fixable Viability Kit (BV510). For the identification of CD4^{+} T cells (C), doublets were excluded followed by a live/dead separation using the Zombie NIR™ Fixable Viability Kit (APC-Cy7). CD3^{+} T cells (APC) were subsequently selected from lymphocytes. Finally, T cells were separated in CD4^{+} (FITC, upper left) and CD8^{+} (PE, lower right) T cells.

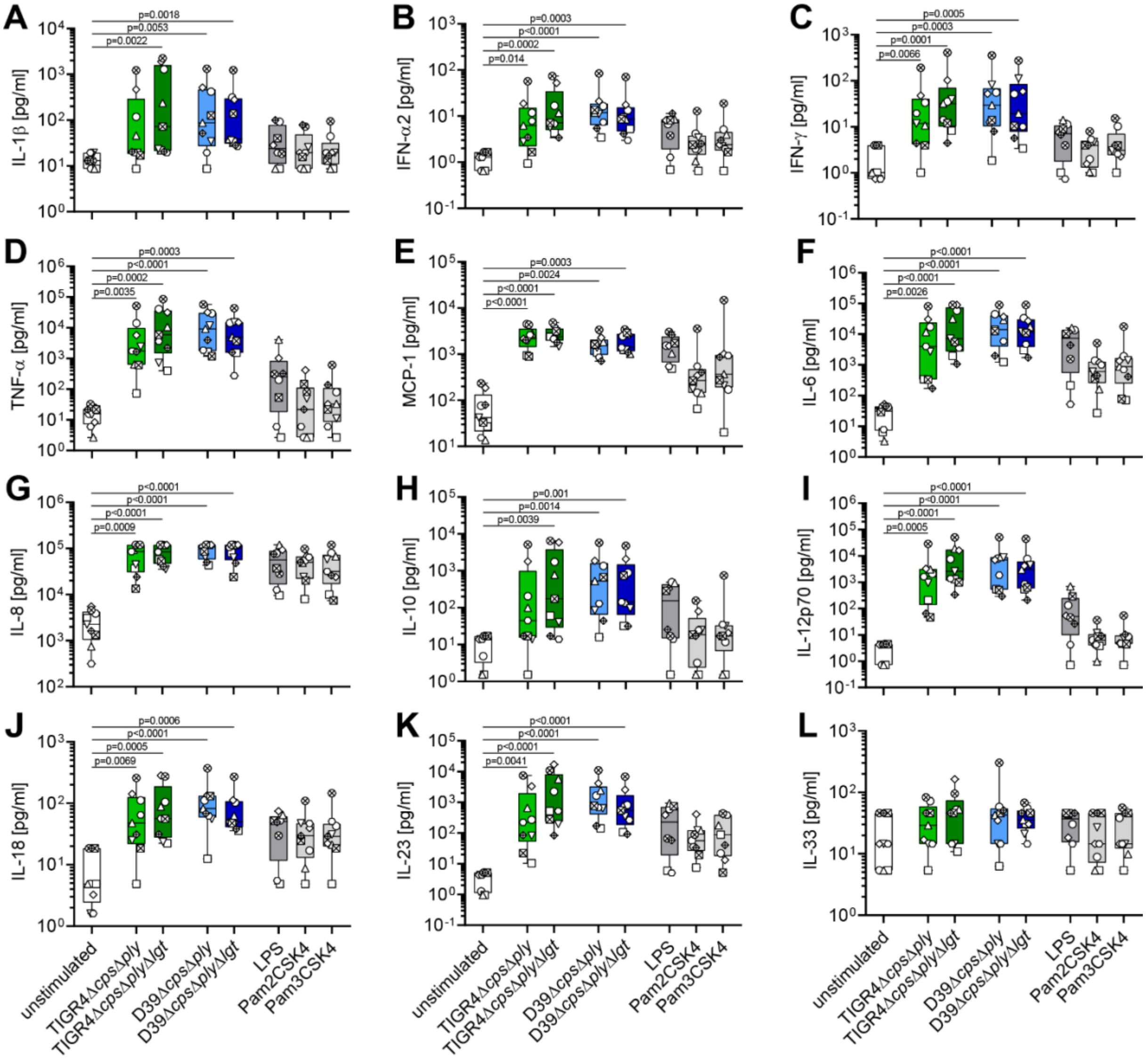

Supplementary Figure 5. Cytokine secretion of human moDCs in response to pneumococcal infection. Cytokine secretion of infected moDCs was evaluated via a multiplex assay by flow cytometry (n=9). The concentration of IL-1β (A), IFN-α2 (B), IFN-γ (C), TNF-α (D), MCP-1 (E), IL-6 (F), IL-8 (G), IL-10 (H), IL-12p70 (I), IL-18 (J), IL-23 (K), and IL-33 (L) was determined in supernatants of (un)infected moDCs. The data are displayed as box plots. Each dot represents the response of one donor. The level of significance was determined using Kruskal-Wallis test with Dunn`s post-test.

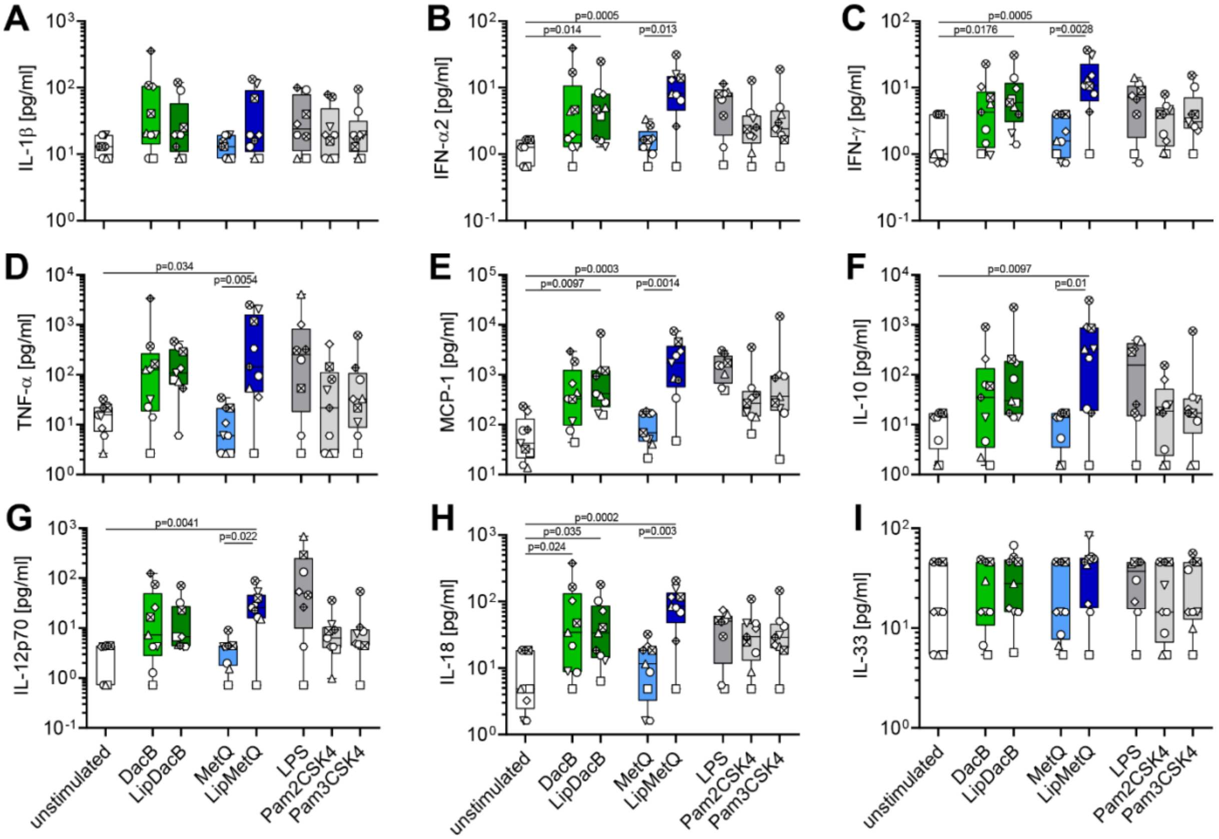

Supplementary Figure 6. Cytokine secretion of moDCs in response to pneumococcal lipidated and non-lipidated lipoproteins. Cytokine secretion of stimulated moDCs was evaluated via a multiplex assay by flow cytometry (n=9). The concentration of IL-1β (A), IFN-α2 (B), IFN-γ (C), TNF-α (D), MCP-1 (E), IL-10 (F), IL-12p70 (G), IL-18 (H), and IL-33 (I) was determined in supernatants of (un)stimulated moDCs. The data are displayed as box plots. Each dot represents the response of one donor. The level of significance was determined using Kruskal-Wallis test with Dunn`s post-test.

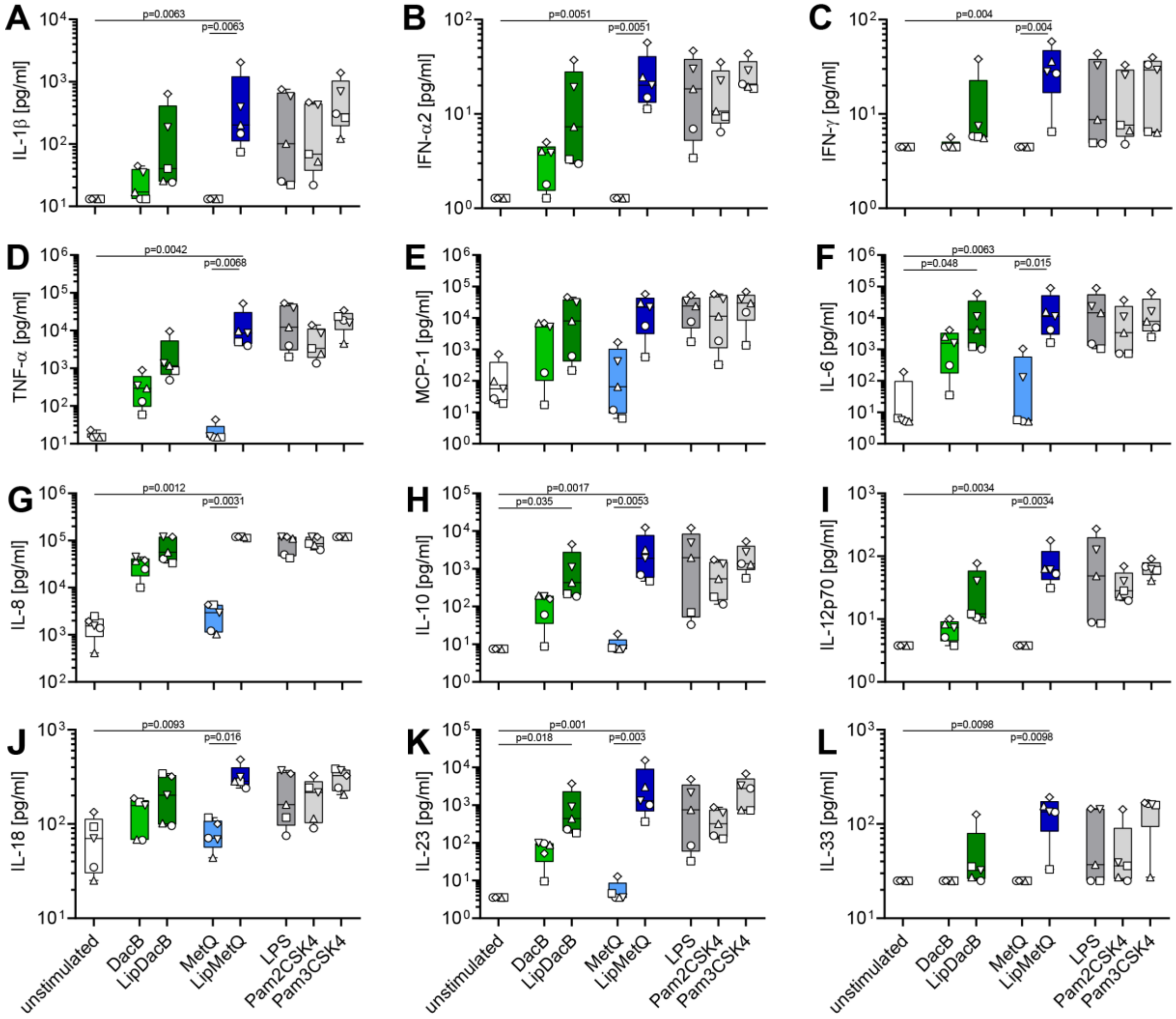

Supplementary Figure 7. Cytokine secretion of MDMs in response to pneumococcal lipidated and non-lipidated lipoproteins. Cytokine secretion of stimulated MDMs was evaluated via a multiplex assay by flow cytometry (n=6). The concentration of IL-1β (A), IFN-α2 (B), IFN-γ (C), TNF-α (D), MCP-1 (E), IL-6 (F), IL-8 (G), IL-10 (H), IL-12p70 (I), IL-18 (J), IL-23 (K), and IL-33 (L) was determined in supernatants of (un)stimulated MDMs. The data are displayed as box plots. Each dot represents the response of one donor. The level of significance was determined using Kruskal-Wallis test with Dunn`s post-test.
